# Dendritic cell cross‐dressing and tumor immunity

**DOI:** 10.15252/emmm.202216523

**Published:** 2022-08-12

**Authors:** Amaia Martinez‐Usatorre, Michele De Palma

**Affiliations:** ^1^ Swiss Institute for Experimental Cancer Research (ISREC), School of Life Sciences Swiss Federal Institute of Technology in Lausanne (EPFL) Lausanne Switzerland; ^2^ Agora Cancer Research Center Lausanne Switzerland; ^3^ Swiss Cancer Center Léman (SCCL) Lausanne Switzerland

**Keywords:** Cancer, Immunology

## Abstract

In addition to direct and cross‐presentation, dendritic cells (DCs) can present tumor antigens (TAs) to T cells via a hitherto poorly understood mechanism called “cross‐dressing.” DC cross‐dressing involves the acquisition of preformed peptide‐major histocompatibility class I/II (p‐MHC) complexes from cancer cells. This process has been documented both in cell culture and in tumor models; may occur via the uptake of tumor‐derived extracellular vesicles or the horizontal transfer of plasma membrane fragments from cancer cells to DCs; and can be enhanced through DC engineering for therapeutic applications. In some experimental contexts, DC cross‐dressing may be essential for productive anti‐tumor immunity, possibly owing to the fact that tumor‐derived p‐MHC complexes encompass the full repertoire of immunologically relevant TAs against which primed cytotoxic T cells can exert their tumoricidal activity.

## Graphical abstract

In this timely Commentary, M. De Palma and A. Martinez‐Usatorre define dendritic cells cross‐dressing and discuss how this phenomenon may be used to enhance anti‐tumor immunity.

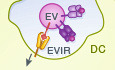

## Acquisition and presentation of tumor antigens by dendritic cells

Dendritic cells (DCs) are specialized myeloid cells with the ability to uptake, process, and present antigens to T lymphocytes; they also generate cytokine and chemokine gradients that regulate lymphocyte trafficking and function (Perez & De Palma, 2019). In both human and mouse system, DCs comprise developmentally distinct subsets encompassing monocyte‐derived DCs (moDC), plasmacytoid DCs, and conventional DCs (cDCs). cDCs can be further resolved into subpopulations, some of which may reflect different functional states rather than true lineages; among these, conventional type I DCs (cDC1) play an integral role in sculpting tumor immunity (Perez & De Palma, 2019; Cabeza‐Cabrerizo *et al*, 2021).

In the context of cancer, DCs can present tumor antigens (TAs) to T lymphocytes through distinct mechanisms. Direct antigen presentation follows the engulfment of tumor‐derived material by the DC. Internalized TAs are processed in the endosomal compartment by lysosomal proteases to generate short peptides, which are loaded onto major histocompatibility class II (MHCII) molecules to form peptide‐MHCII (p‐MHCII) complexes that are transported to the plasma membrane for presentation; this form of antigen presentation leads to activation of CD4^+^ T cells, which can engage other cellular components of the immune system (Perez & De Palma, 2019). DCs may also present TAs on MHCI molecules; this mode of antigen presentation is called cross‐presentation and is conducive to cross‐priming of naïve CD8^+^ T cells. Following internalization, TAs may be either exported to the cytosol from the endosome/phagosome and processed by the proteasome into peptides that are loaded onto MHCI, or processed by lysosomal proteases and loaded onto MHCI directly in the endosome. In both cases, cross‐presentation by DCs cross‐primes CD8^+^ T cells that are specific for MHCI‐restricted TAs. Among DC subsets, cDC1 have superior cross‐presentation capacity and, in fact, cross‐presentation by cDC1 was shown to be necessary for CD8^+^ T‐cell‐mediated tumor rejection in multiple studies (Perez & De Palma, 2019; Cabeza‐Cabrerizo *et al*, 2021).

**Figure 1:**
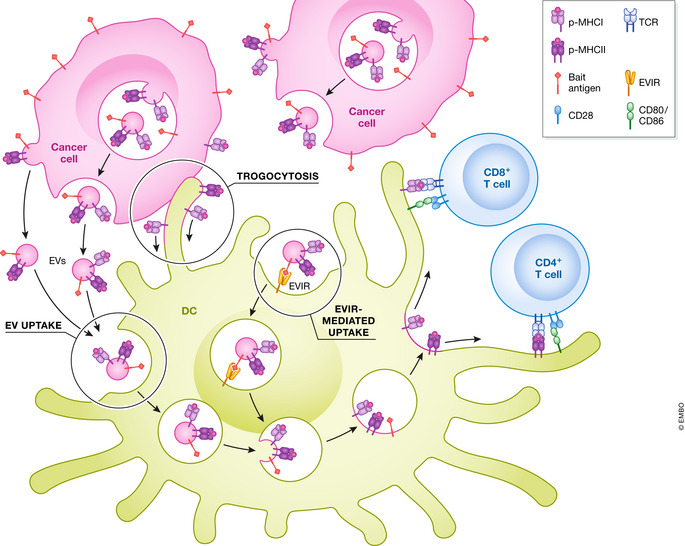
Exploring and exploiting dendritic cell cross‐dressing in cancer Cancer cells may deploy preformed p‐MHC complexes to DCs through EVs or via trogocytosis, the horizontal transfer of membrane fragments (left). Following acquisition of EVs or membrane fragments, cancer‐cell‐derived p‐MHC complexes may either be internalized and recycled to the DC's plasma membrane, or directly displayed on the DC's plasma membrane. In both instances, p‐MHC complexes can directly prime CD4^+^ and CD8^+^ T cells. According to a DC engineering strategy, DCs may be armed with a chimeric receptor, called EVIR, which can enhance the uptake of tumor‐derived EVs/membranes, thereby enhancing DC cross‐dressing (right). Endogenous p‐MHC complexes are not illustrated in the figure. DC, dendritic cell; EV, extracellular vesicle; EVIR, extracellular vesicle‐internalizing receptor; p‐MHC, peptide‐major histocompatibility class I/II; TRC, T‐cell receptor.

In addition to direct presentation and cross‐presentation, DCs can present TAs to T cells via a hitherto less known and largely neglected mechanism, called “cross‐dressing” (Fig 1). This involves the acquisition of preformed p‐MHCI complexes from cancer cells, which may occur through the uptake of tumor‐derived extracellular vesicles (EVs) or via a trogocytosis‐like mechanism encompassing the horizontal transfer of plasma membrane fragments from cancer cells to DCs. Both mechanisms can lead the DC to display functional p‐MHCI complexes capable of activating tumor‐specific T cells (Zeng & Morelli, 2018; Perez & De Palma, 2019).

## Early evidence for dendritic cell cross‐dressing

Early evidence suggested that DCs can directly acquire functional, preformed p‐MHC complexes from heterologous cells. Russo *et al* (2000) observed direct transfer of human MHCI molecules, called human leukocyte antigens (HLAs), from melanoma cells to moDCs in co‐culture experiments. Indeed, moDCs isolated from an HLA‐A2‐negative individual became loaded with HLA‐A2 after co‐culture with HLA‐A2^+^ melanoma cells; moreover, HLA‐A2‐specific cytotoxic CD8^+^ T cells were able to kill moDCs exposed to an HLA‐A2‐positive melanoma line, whereas moDCs co‐cultured with HLA‐A2‐negative melanoma cells were not recognized. These results were among the first to illustrate the horizontal transfer of functional p‐HLA complexes from cancer cells to DCs. Six years later, Dolan *et al* (2006a) introduced the term “cross‐dressing” to denote the “generation of p‐MHC complexes […] and their subsequent transfer to DCs, which then present the intact, unprocessed complexes to T lymphocytes”.

Collectively, a number of early studies (Patel *et al*, 1999; Russo *et al*, 2000; Théry *et al*, 2002; Herrera *et al*, 2004; Dolan *et al*, 2006a, 2006b) indicated that (i) DCs can acquire autologous or allogenic p‐MHC complexes from other antigen‐presenting cells or cancer cells; (ii) both p‐MHCI and p‐MHCII complexes can cross‐dress DCs; (iii) the endogenous MHC/HLA machinery may be dispensable for DCs to activate T cells through cross‐dressing and (iv) both secreted EVs and membrane transfer via cell‐to‐cell contacts provide biologically relevant sources of preformed p‐MHC complexes for DC cross‐dressing. The significance of DC cross‐dressing for immunological processes underlying viral infections, transplant rejection and tolerance, and cancer is being increasingly appraised (Zeng & Morelli, 2018).

## Dendritic cell cross‐dressing in cancer: causes and consequences

Cancer cells may express MHCI molecules, display p‐MHCI complexes, and activate antigen‐experienced CD8^+^ T cells, but are generally not naturally equipped to present MHCII‐restricted antigens that would directly prime CD4^+^ T cells. Live cancer cells engineered to express MHCII along with co‐stimulatory molecules could, in principle, provide a vaccination strategy to directly prime naïve CD4^+^ T cells against the parental tumor. In a pioneering study, Dolan *et al* showed that endogenous DCs are required for the efficacy of a live cancer vaccine based on MHCII‐expressing sarcoma cells. Indeed, whereas the live vaccine induced lethal ascites in CD11c‐DTR mice in which DCs were transiently ablated, it failed to do so in mice with an intact DC pool, indicating that MHCII‐expressing cancer cells could not directly prime CD4^+^ T cells. Critically, cell culture studies demonstrated that DCs acquired preformed p‐MHCII complexes from dying cancer cells through cell‐to‐cell contacts and membrane transfer, which was conducive to the activation of CD4^+^ T cells (Dolan *et al*, 2006a).

In a related study (Dolan *et al*, 2006b), MHCI‐proficient DAP cells (an immortalized fibroblast cell line) were found to transfer preformed p‐MHCI complexes to DCs, which in turn activated naïve CD8^+^ T cells in cell culture. By vaccinating mice with mixtures of DAP cells—either live or dead, and expressing a surrogate antigen (ovalbumin, OVA) with or without relevant MHCI molecules—the authors demonstrated that DC cross‐dressing mediated by dead cells and their p‐MHCI complexes was *necessary* albeit not sufficient to maximally activate OVA‐specific CD8^+^ T cells *in vivo* (Dolan *et al*, 2006b). While using sophisticated and somewhat artificial immunological assays, these landmark studies (Dolan *et al*, 2006a, 2006b) emphasized the potential significance of DC cross‐dressing for anti‐tumor immunity.

Recent work has begun to explore the relevance and mechanisms of DC cross‐dressing in more physiological tumor models. Das Mohapatra *et al* (2020) used mice inoculated with either live or irradiated EG7 lymphoma cells, which were previously labeled with carboxyfluorescein succinimidyl ester (CFSE) to mark cytosolic proteins or PKH26 to mark cell membranes. They found that CD8α^+^ DCs, a population of lymph node‐resident cDC1, acquired both CFSE and PKH26 from irradiated cancer cells but only PKH26 from live cancer cells. These results suggested that live cancer cells were resistant to phagocytosis but could transfer membranes to DCs through a mechanism potentially involving trogocytosis. Further studies indicated that live EG7 cells cross‐dressed CD8α^+^ DCs with functional p‐MHCI antigens in tumor‐draining lymph nodes. This was shown by using a mutant mouse able to load the OVA peptide SIINKEKL on MHCI but unable to present the resulting p‐MHCI complex to OVA‐specific T cells. When challenged with live EG7 cells (which expressed wild‐type MHCI and OVA), mutant mice retained the ability to prime adoptively transferred OVA‐specific T cells, suggesting that DC cross‐dressing could rescue endogenously impaired antigen presentation to T cells. Interestingly, live cancer cells could immunize mice more efficiently than irradiated/dead cancer cells in tumor rechallenge experiments. The authors speculated that whereas live cancer cells were resistant to phagocytosis but could efficiently cross‐dress CD8α^+^ DCs, irradiated/dead cancer cells were phagocytosed by a broad population of myeloid cells, including macrophages, which may have instigated tolerogenic and immunosuppressive mechanisms that limited cross‐dressing‐induced anti‐tumor immunity (Das Mohapatra *et al*, 2020).

The insightful results of Dolan *et al* (2006a, 2006b) and Das Mohapatra *et al* (2020) raise a number of questions. The relative contributions of live versus dead cancer cells, or uptake of EVs/apoptotic bodies versus direct membrane transfer, to DC cross‐dressing remain unclear: while the former studies emphasized the requisite role of dead‐cell‐derived p‐MHC complexes, the latter study largely implicated trogocytosis from live cancer cells. Identifying the exact source of preformed p‐MHC complexes for DC cross‐dressing *in vivo* may prove challenging, as live cancer cells undergo cell death also in progressing tumors, the extent of which is tumor type and size dependent. Also, recent data indicate that cancer cells secrete heterogeneous populations of EVs, whose biogenesis and molecular composition vary with the tumor type (Beltraminelli *et al*, 2021). In this regard, Squadrito *et al* showed that tumor‐derived EVs provide an effective source of preformed p‐MHCI complexes for DC cross‐dressing, both in cell culture and in mouse allograft models. Moreover, they showed that EV‐mediated TA transfer to DCs and the ensuing priming of TA‐specific CD8^+^ T cells are strictly dependent on cross‐dressing, whereas cross‐presentation and T‐cell cross‐priming have negligible roles (Squadrito *et al*, 2018).

Two recent studies demonstrated that the requirement of DC cross‐dressing for T‐cell priming and anti‐tumor immunity in syngeneic mouse tumors is DC type and tumor‐model dependent (Duong *et al*, 2022; MacNabb *et al*, 2022). Duong *et al* found that type‐I interferon (IFN)‐stimulated cDC2 more efficiently cross‐dress with preformed p‐MHCI complexes than cDC1. In immunogenic MC57‐SIY tumors, which constitutively express type‐I IFN, tumor regression was sustained by both cross‐dressed cDC2 and cross‐presenting cDC1. Conversely, in progressing MC38‐SIY tumors that lacked cross‐dressed cDC2, cross‐presenting cDC1 failed to orchestrate productive anti‐tumor immunity. Remarkably, exogenously administered type‐I IFN could rescue anti‐tumor immunity in MC38‐SIY tumors, leading to their regression, also in mice genetically modified to lack cDC1 cells (Duong *et al*, 2022). In apparent contradiction with the aforementioned results, MacNabb *et al* found that intra‐tumoral cDC1 became more efficiently cross‐dressed with p‐MHCI than cDC2. Adding to the complexity, they showed that p‐MHCI cross‐dressing is *sufficient* to prime tumor‐specific CD8^+^ T cells in the C1498 lymphoma but not in the 1969 sarcoma model (MacNabb *et al*, 2022). Therefore, the requirement of DC cross‐dressing for tumor‐specific T‐cell priming may be context dependent.

## Therapeutic implications and concluding remarks

In the last 20 years, increasing data have corroborated initial evidence that DCs (or specific subsets thereof) can acquire functional, preformed p‐MHC complexes from cancer cells to prime both CD4^+^ and CD8^+^ T cells against cancer. Intriguingly, while conventional cross‐presentation was dispensable, DC cross‐dressing was essential for anti‐tumor immunity in certain experimental contexts. This may be due to the fact that tumor‐derived p‐MHCI complexes potentially encompass the full repertoire of immunologically relevant TAs against which primed cytotoxic T cells can exert their tumoricidal activity. Thus, therapeutic interventions that encourage DC cross‐dressing may hold promise for stimulating immune responses against progressing tumors.

To enhance DC cross‐dressing, Squadrito *et al* (2018) generated a platform of chimeric receptors, called EV‐internalizing receptors (EVIRs), which can enforce moDC uptake of EVs and other membrane fragments specifically derived from cancer cells. Through an scFv antibody domain, the EVIR recognizes a molecule (called bait antigen) that is displayed on the surface of EVs and other membranes shed by cancer cells (Fig 1). The authors found that expression of the EVIR magnified moDC cross‐dressing with tumor‐derived p‐MHCI complexes; in particular, EVIR‐induced DC cross‐dressing was associated with internalization and recycling of functional p‐MHCI complexes from adsorbing tumor‐derived EVs. This process encompassed macropinocytosis and sorting of EVs to EEA1^+^ early endosomes, which was followed by sorting of membrane/p‐MHCI complexes to RAB11^+^ recycling endosomes and, finally, to the plasma membrane of the moDC (Squadrito *et al*, 2018). Future studies should explore the potential of cDC‐based EVIR vaccines, especially in light of recent findings arguing that cDC2 and cDC1 get more proficiently cross‐dressed than other DC subtypes in syngeneic tumors (Duong *et al*, 2022; MacNabb *et al*, 2022).

The immunogenic potential of DC cross‐dressing should be increased by treatments that abate tolerogenic antigen presentation sustained by tumor‐associated macrophages (TAMs). The elimination of TAMs using colony‐stimulating factor‐1 receptor antibodies may, therefore, contribute to remove a population of antigen‐presenting cells with immunosuppressive capacity. Moreover, both anti‐angiogenic therapy and certain chemotherapeutic agents can enhance endogenous DC maturation and promote anti‐tumor immunity. It remains to be seen whether such treatments have, *per se*, the ability to directly stimulate functional DC cross‐dressing with TAs.

## Author contributions

**Michele De Palma:** Conceptualization; funding acquisition; writing – original draft; writing – review and editing. **Amaia Martinez‐Usatorre:** Conceptualization; writing – original draft; writing – review and editing.

## Disclosure and competing interests statement

MDP is an inventor on a patent application filed by EPFL (WO2017134100A1) on engineered dendritic cell vaccines. MDP serves on the scientific advisory board of EVIR Therapeutics, a start‐up focused on the development of engineered dendritic cell vaccines for cancer therapy.
